# Construction of nanostructures for selective lithium ion conduction using self-assembled molecular arrays in supramolecular solids

**DOI:** 10.1080/14686996.2017.1366816

**Published:** 2017-08-30

**Authors:** Makoto Moriya

**Affiliations:** ^a^ Faculty of Science, Department of Chemistry, Shizuoka University, Shizuoka, Japan

**Keywords:** Ion conduction, supramolecules, self-assembly, molecular crystals, lithium, solid electrolytes, 20 Organic and soft materials (colloids, liquid crystals, gel, polymers), 101 Self-assembly / Self-organized materials, 206 Energy conversion / transport / storage / recovery, 501 Chemical analyses, 504 X-ray / Neutron diffraction and scattering

## Abstract

In the development of innovative molecule-based materials, the identification of the structural features in supramolecular solids and the understanding of the correlation between structure and function are important factors. The author investigated the development of supramolecular solid electrolytes by constructing ion conduction paths using a supramolecular hierarchical structure in molecular crystals because the ion conduction path is an attractive key structure due to its ability to generate solid-state ion diffusivity. The obtained molecular crystals exhibited selective lithium ion diffusion via conduction paths consisting of lithium bis(trifluoromethanesulfonyl)amide (LiTFSA) and small molecules such as ether or amine compounds. In the present review, the correlation between the crystal structure and ion conductivity of the obtained molecular crystals is addressed based on the systematic structural control of the ionic conduction paths through the modification of the component molecules. The relationship between the crystal structure and ion conductivity of the molecular crystals provides a guideline for the development of solid electrolytes based on supramolecular solids exhibiting rapid and selective lithium ion conduction.

## Graphical abstract

**Figure:**
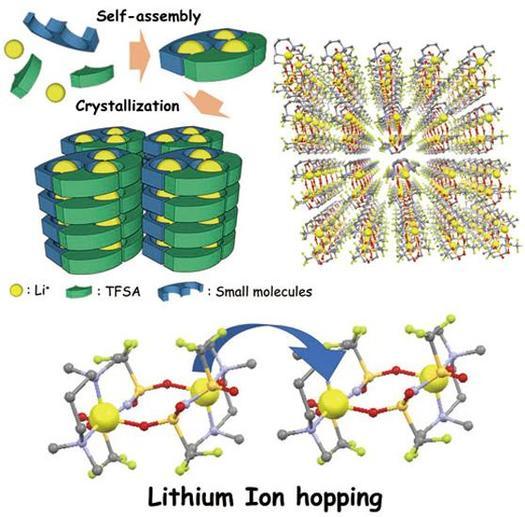

## 1. Introduction

The construction of supramolecular assemblies and control of their hierarchical structures has attracted considerable attention because of their potential to allow the development of molecule-based materials [1–4]. In particular, the application of ordered molecular assemblies in supramolecular solid to solid state ionics is an attractive method for the fabrication of innovative electrochemical devices [5–11]. One of the most important functions of such supramolecular solids is lithium ion conductivity, which would allow their applications in all-solid batteries, providing both flexibility and safety [12–21].

To create solid electrolytes using such molecular assemblies, it is necessary to understand the relationship between their structure and function. Crystallization seems to be a feasible method of constructing conduction paths for lithium ion diffusion since molecular crystals (MCs) possess three-dimensional (3D) ordered arrangements of component molecules in their crystal lattices. Furthermore, the 3D ordered structures are easily altered by modifying the component molecules.

However, MCs have received relatively little attention as potential solid electrolytes compared to amorphous polymers with lithium ion conductivity [22–33]. One of the reasons for this is the difficulty in fabricating conduction paths to allow ion diffusion in MCs. In fact, only a few types of MCs with ion conduction paths have been reported, which have cylindrical structures consisting of poly(ethylene oxide) (PEO) [22,26–31], crown ethers [32,33], or glymes [23–25] with lithium ions. Furthermore, the relationship between the structure of the conduction paths and the conductivity of ions through the MCs is still unclear because of the lack of material design guidelines. However, these reports indicate that the ion conductivity of the MCs, which is not sufficient for practical applications at present, is greatly affected by the structure of the conduction paths.

**Figure 1.**
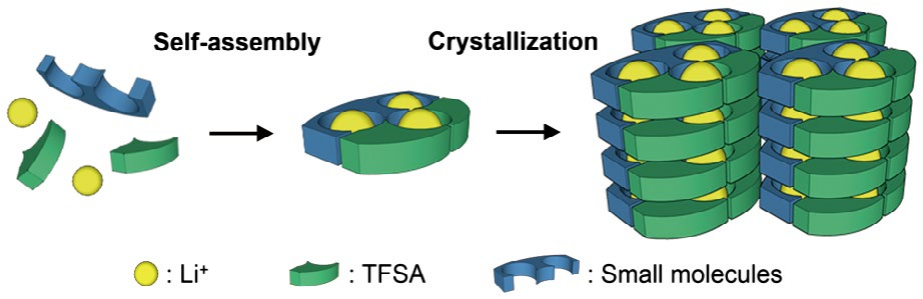
Schematic diagram of the development of ion conduction paths using supramolecular assemblies.

The development of a method to form and control the structure of ion conduction paths is essential for the use of MCs as solid electrolytes. To achieve this goal, we have focused on hierarchical supramolecular structures provided by the self-assembly of small molecules and counter anions of lithium salts to obtain MCs with lithium ion conductivity [34–37]. In our strategy, ion channels suitable for lithium ion conduction consisting of small molecules and lithium salts are formed in the frameworks of the self-assembled structures. Then, ion conduction paths are constructed via stacking of the channel structures using crystallization (Figure 1). MCs show diversity both in terms of the structure of the component molecules and the arrangement of these molecules. This allows precise control of the three-dimensional ordered structure at the molecular level, giving a high degree of freedom for the design of conduction paths for rapid ion diffusion.

We also attempted to achieve selective lithium ion conduction through molecular crystalline electrolytes by suppressing anion diffusion. In this work, we employed the bis(trifluoromethanesulfonyl)amide anion, [N(SO_2_CF_3_)]_2_^−^ (TFSA), for this purpose. TFSA anion possesses not only sulfonyl groups capable of interacting with lithium ions via the oxygen atom, but also the electron withdrawing trifluoromethyl group, which should result in high ionicity [38–41]. Furthermore, TFSA anion possesses conformational flexibility derived from its linear structure that enables it to interact with lithium ions in various coordination geometries. These characteristics make the TFSA anion suitable for use as a component in conduction paths for fast and selective ion migration through MCs.

**Figure 2.**
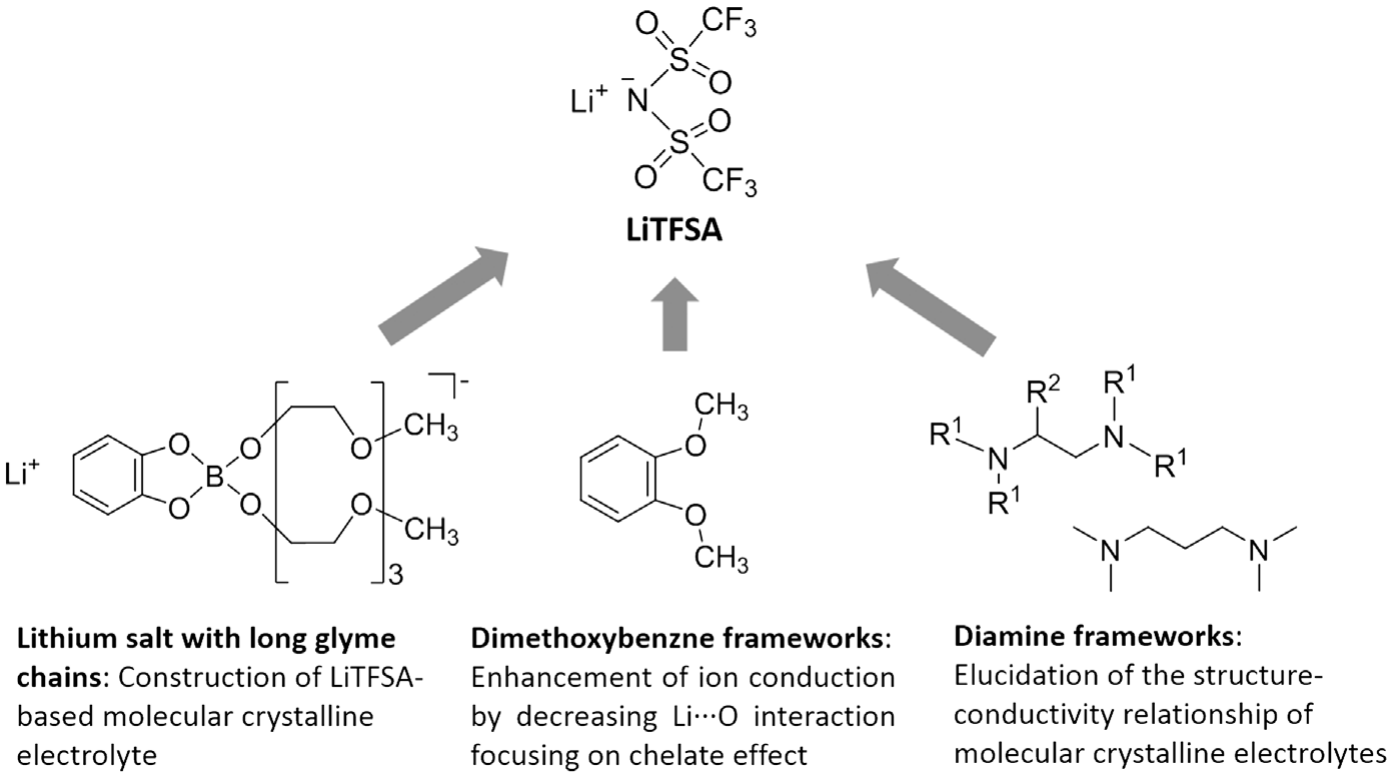
The designing concept of molecular crystalline electrolytes.

From this viewpoint, we have investigated the development of molecular crystalline materials for solid electrolytes. First, we synthesized a LiTFSA-based molecular crystal and evaluated its ion conductivity in the crystalline state using lithium borate having long glyme chains to form channel structures as a component unit. Second, we attempted to increase the ion conductivity of MCs focusing on the reduction of chelate effect from glyme units to lithium centers. Third, we tried to clarify the structure-conductivity relationship of molecular crystalline electrolytes by the precise structural control of MCs using diamine framework (Figure 2). Herein we review the synthesis of novel MCs as candidates for the new class of solid electrolyte [34–37]. We also discuss the correlation between the structures and properties of the obtained compounds based on systematic structural control of the MCs to establish material design guidelines for unconventional solid electrolytes.

## 2. MCs with lithium borate having glyme chains 

We initially designed a novel starting compound, Li[B(C_6_H_4_O_2_)(O(CH_2_CH_2_O)_3_CH_3_)_2_] (compound **1**), using glyme units to form channel structures, then investigated the synthesis of novel MCs with ion conduction paths by combining LiTFSA and compound **1** [37]. An aromatic ring was introduced to compound **1** to enhance crystallization via the use of intermolecular interactions. An anionic form of boron was selected to connect the aromatic ring to the glyme chains and act as a counter anion for lithium ions to achieve selective lithium ion diffusion.

**Figure 3.**
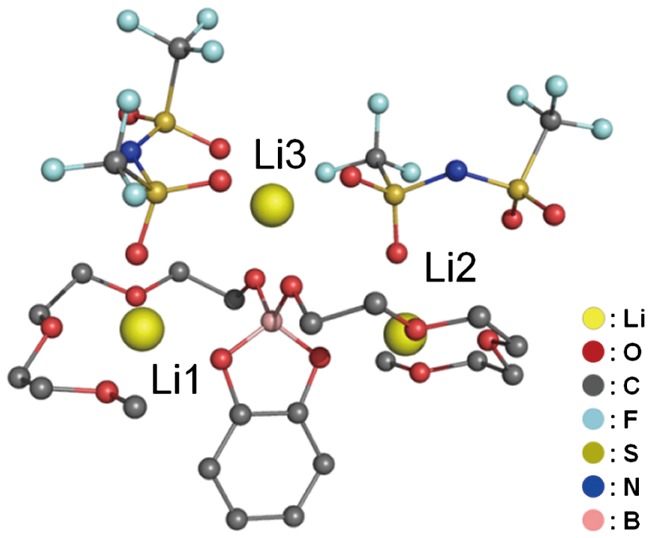
Crystal structure of compound **2** determined from the powder X-ray diffraction (XRD) pattern at 100 K. (Reproduced from [37] with permission from John Wiley and Sons.).

The reaction of [LiB(OCH_3_)_4_] or [LiB(OCH_2_CH_2_OCH_3_)_4_] with two equivalents of triethylene glycol monomethyl ether followed by the addition of an equimolar amount of catechol yielded compound **1** as a colorless viscous liquid. The reaction between compound **1** and LiTFSA proceeded in 1:2 molar ratio to give the trilithium compound Li_3_[B(C_6_H_4_O_2_)(O(CH_2_CH_2_O)_3_CH_3_)_2_][N(SO_2_CF_3_)_2_]_2_ (compound **2**). Compound **2** was a white crystalline powder, and its structure was determined from a synchrotron powder diffraction experiment carried out at the Spring-8 BL02B2 beam line using an imaging plate as a detector [42] (Figure 3).

**Figure 4.**
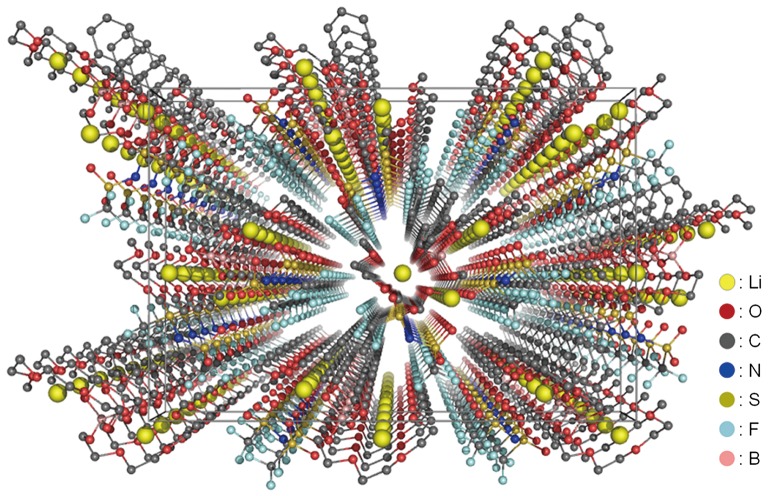
The packing view of compound **2** along the *b*-axis determined from the XRD pattern at 100 K. (Reproduced from [37] with permission from John Wiley and Sons.).

As shown in Figure 4, nano-structured units corresponding to ion channels were presented in compound **2** because of the electrostatic interactions between the lithium ions and the oxygen atoms of the TFSA anions and glyme chains. Compound **2** possesses three types of Li ions in its nano-structure, denoted as Li1, Li2 and Li3 for convenience.

Both Li1 and Li2 are present in the channel structures formed by the glyme chains. These lithium ions are coordinated by five oxygen atoms, with four being from the glyme moiety of compound **1** and one being from the TFSA anion. These lithium ions form square pyramidal coordination structures. In contrast, Li3, which is outside the channel, takes the form of a hindered trigonal bipyramidal structure. A view of the packing of compound **2** along the b-axis clearly displays the formation of conduction paths with a one-dimensional arrangement of the lithium ions. These conduction paths are constructed by the stacking of the channel structures of compound **2**.

To investigate the thermal stability of the conduction paths of compound **2**, the variable-temperature XRD data were collected from −173 to 127 °C and the differential scanning calorimetry (DSC) analysis was performed. The peak shape, width and intensity of the 127 °C data are almost identical to those from the −173 °C data. Furthermore, the DSC curve of compound **2** shows only one endothermic peak attributed to melting at 165 °C. This result indicates that the characteristic structure of the ion conduction paths of compound **2** is maintained even at the high temperature.

**Figure 5.**
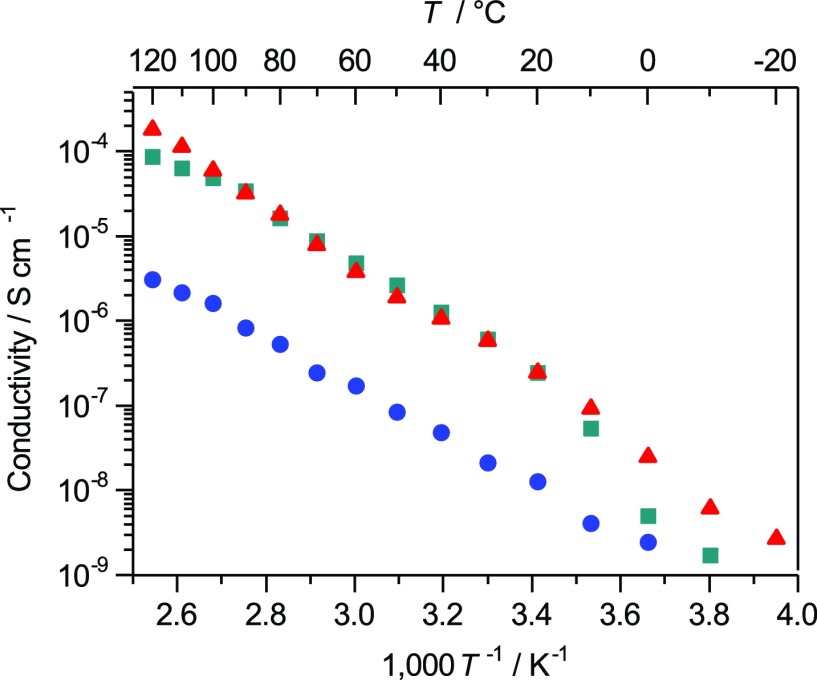
Ion conductivities of compound **2** (blue), compound **1**+2.5LiTFSA (red) and compound **1**+1.8LiTFSA (green). (Adapted from [37] with permission from John Wiley and Sons).

Considering these structural features, the ion conductivity of compound **2** in the crystalline state was measured by the AC impedance method. A powder sample of compound **2** was pressed into a disk and placed between a pair of stainless steel disks in a measurement cell with this process being performed in an argon-filled glove box to avoid contamination by moisture. Although the conductivity of compound **2** was quite low at 10^−8^ S cm^−1^ at room temperature, we confirmed that ion conduction occurred through the crystal lattices of compound **2** (Figure 5). The Arrhenius plot for compound **2** is linear, indicating that ion conduction takes place via a hopping mechanism. The AC impedance data showed a well-defined semicircle and a low-frequency spike, similar to the previously reported AC impedance results for polymer electrolytes. These results indicate that the grain boundary resistance of these MCs was relatively small.

We found that the use of TFSA anions as a building block for MCs not only led to the construction of conduction paths, but also enabled the selective conduction of lithium ions. This was confirmed by the measurement of lithium transport numbers (*t*_Li+_). The value of *t*_Li+_ was determined to be 0.9 indicating that compound **2** behaved as a single-ionic conductor. Since the anionic borate units of compound **1** and the TFSA moieties formed channel structures via intramolecular Li–O interactions, anion conduction was suppressed in compound **2**. This led to the high *t*_Li+_ value, in addition to the formation of unconventional conduction paths.

The introduction of defects to the crystal structure of compound **2** was also attempted in order to increase the ion conductivity. To achieve this, we changed the molar ratio of compound **1** to LiTFSA from its initial value of 1:2. The formation of defects in these compounds was supported by DSC and XRD measurements. The DSC curves of these compounds were quite similar to that of compound **2**, although they showed slightly lower melting points. The XRD patterns were found to show almost identical diffraction patterns to that of compound **2**. These results showed that certain amounts of defects could be introduced to the crystal structure of compound **2** while maintaining the structure of the ion conduction paths by altering the ratio of LiTFSA to compound **1**. The compounds given by the addition of 1.8 or 2.5 equivalents of LiTFSA to compound **1** exhibited ion conductivities that were approximately 10 times higher than that of compound **2** (Figure 5). Lithium ions in these compounds participated in ion hopping via both the lattice defects and the conduction paths, resulting in improved ion conductivity.

## 3. MCs with 1,2-dimethoxybenzene frameworks

As mentioned above, lithium ion conduction occurred through compound **2** via ion hopping. This process is induced by the repeated cleavage and formation of electrostatic interactions between lithium ions and the oxygen atoms in the ether moiety. This mechanism suggests that the ion conductivity could be enhanced by weakening the Li–O interactions. In fact, the crystal structure of compound **2** indicates that the lithium ions were strongly chelated to the relatively long glyme chains. Thus, we attempted to reduce the chelating effect of the channel structure by substituting the tetradentate ligand with two bidentate ligands.

**Figure 6.**
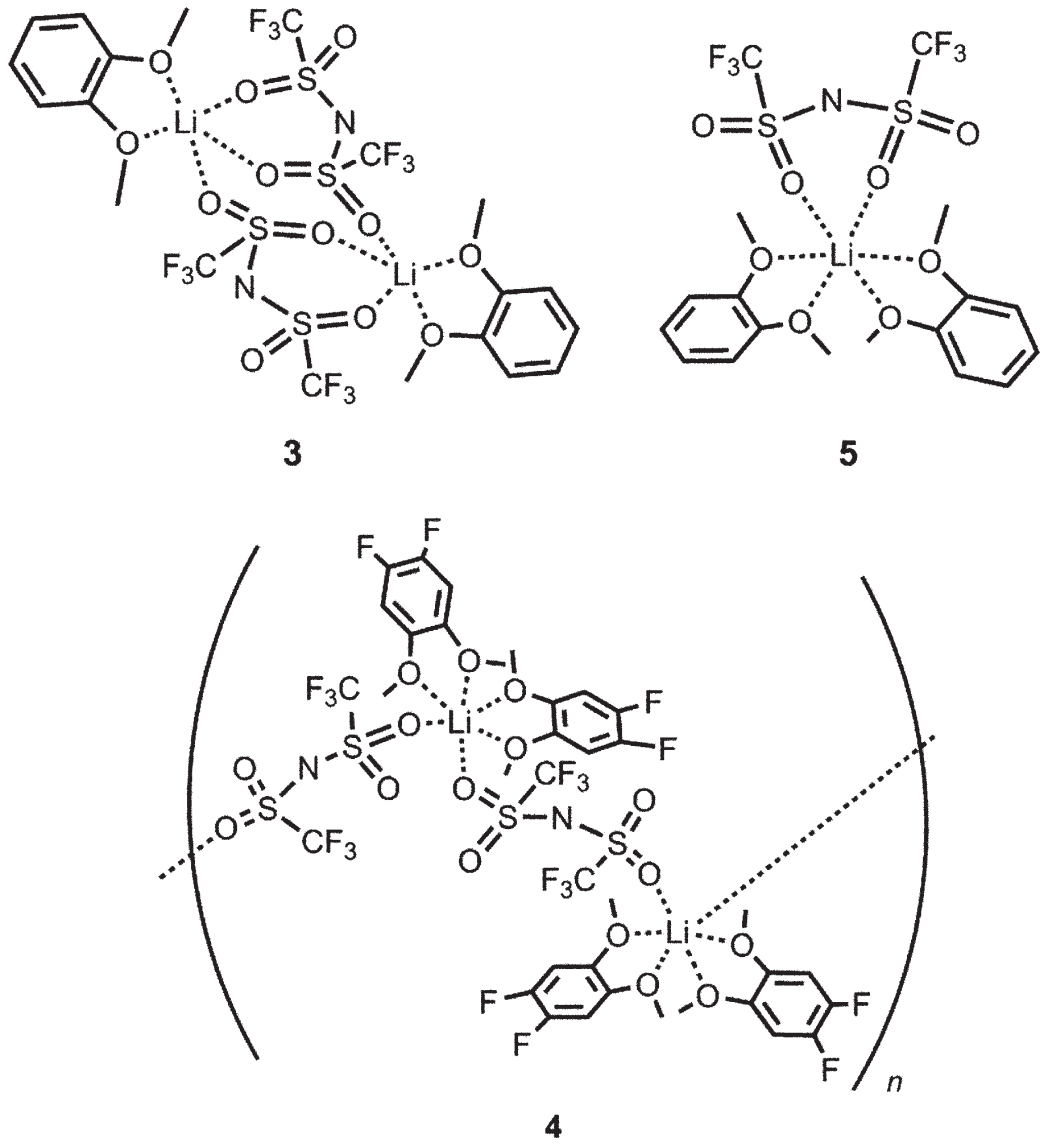
Molecular structures of compounds **3–5**.

To achieve this, 1,2-dimethoxybenzene frameworks were employed as bidentate ligands in MCs. The resulting MCs [Li(N(SO_2_CF_3_)_2_)(C_6_H_4_(OCH_3_)_2_)_2_] (compound **3**) and [Li((NSO_2_CF_3_)_2_)(C_6_F_2_H_2_(OCH_3_)_2_)_2_] (compound **4**) were synthesized by heating LiTFSA with two equivalents of 1,2-dimethoxybenzene or 1,2-difluoro-4,5-dimethoxybenzene, respectively [36] (Figure 6). The analogous MC [Li((NSO_2_CF_3_)_2_)(C_6_H_4_(OCH_3_)_2_)] (compound **5**) was also obtained by the reaction of LiTFSA with an equimolar amount of 1,2-dimethoxybenzene [34].

The DSC curves of compounds **3**, **4** and **5** exhibited single endothermic peaks attributed to melting, at 57.7, 47.8 and 94.7°C, respectively. The melting points of these compounds were higher than that of a reported compound [Li(N(SO_2_CF_3_)_2_)(H_3_COCH_2_CH_2_OCH_3_)_2_] containing 1,2-dimethoxyethane, which melts at approximately 20°C [43]. These results show that the introduction of aromatic rings into the bidentate ligands resulted in an increase in the melting point.

**Figure 7.**
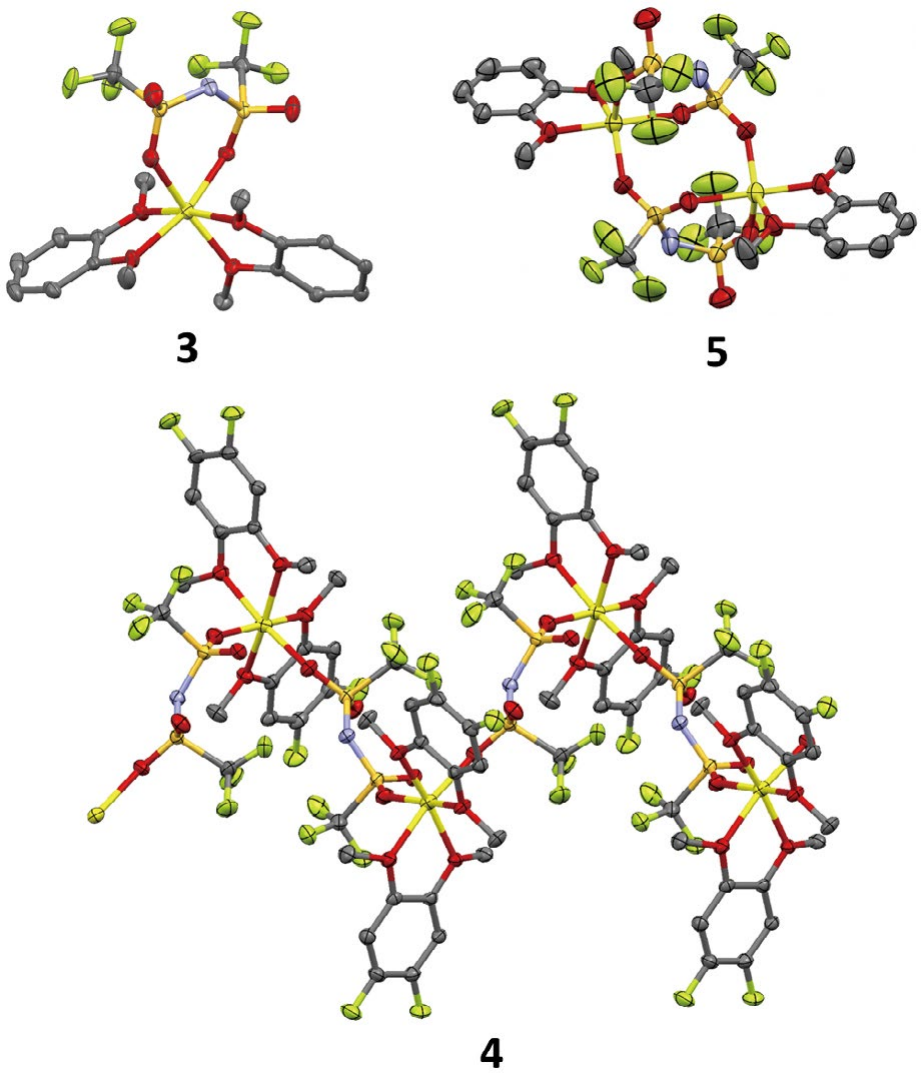
Crystal structures of compounds **3–5** determined from single-crystal X-ray diffraction study at -120 °C. (Li: yellow, C: gray, N: blue, O: red, F: green, S: dark yellow. Hydrogen atoms have been omitted for clarity.) (Adapted from [34] and [36] with permission from John Wiley and Sons and Elsevier.).

**Figure 8.**
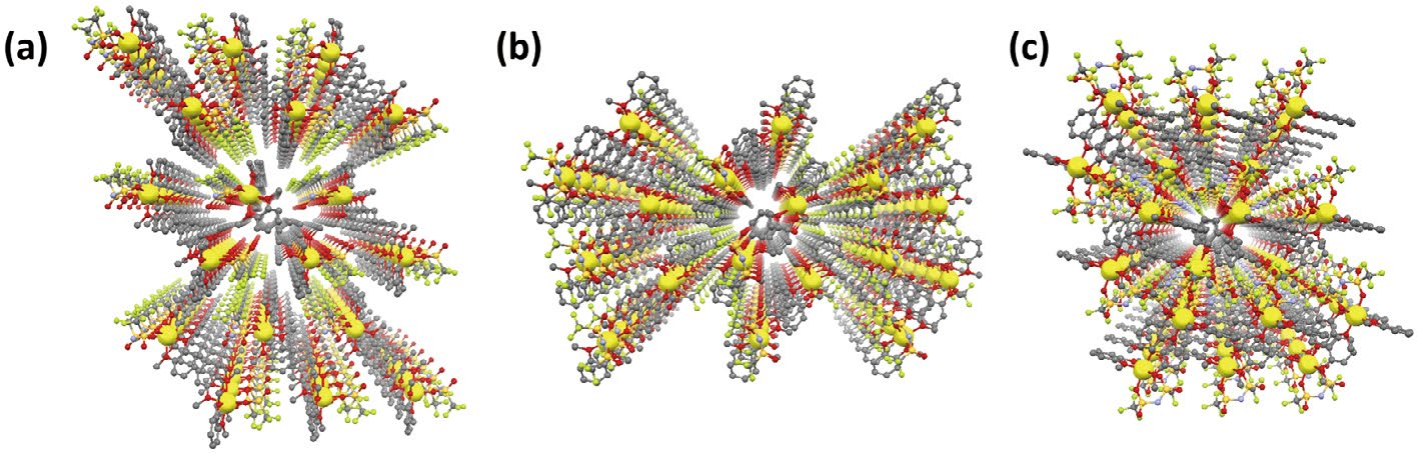
Packing view of compound **3** determined from single-crystal X-ray diffraction study at -120 °C. (a) along the *a*-axis, (b) along the *b*-axis, (c) along the *c*-axis (Li: yellow, C: gray, N: blue, O: red, F: green, S: dark yellow. Hydrogen atoms have been omitted for clarity.) (Adapted from [36] with permission from John Wiley and Sons.).

The crystal structures of compounds **3–5** were revealed by single-crystal X-ray diffraction studies (Figure 7). Compound **3** was a monolithium compound containing two 1,2-dimethoxybenzene molecules and a TFSA anion, which coordinated to the lithium ions in a chelated mode via the oxygen atoms. Compound **4** was afforded as a coordination polymer due to the bridging coordination of the TFSA anions. The molecular structures of compounds **3** and **4** were completely different, although the coordination environments of the lithium ions in these compounds were almost identical. Both compounds **3** and **4** possessed six-coordination lithium centers in octahedral structures with similar Li–O and Li–N distances (ca. 2.10 Å). This result indicates that in this case, the electron withdrawing character of the fluorine groups in compound **4** had only a small effect on the coordination environment of the lithium centers. The structural differences between compounds **3** and **4** were probably induced by the steric bulkiness of 1,2-difluoro-4,5-dimethoxybenzene compared to that of 1,2-dimethoxybenzene. The 3D ordered arrangements of lithium ions were confirmed from the packing views of these compounds although compound **2** and reported PEO-based crystalline polymer electrolytes [26,28,29,31] possess one-dimensional conduction paths (Figure 8). The shortest lithium–lithium distance in compound **3** was calculated to be 7.93 Å, whereas in compound **4** it was estimated to be 8.45 Å. This corresponds to the length between two lithium centers bridged by a TFSA anion.

Compound **5** was a dilithium compound with trigonal bipyramidal lithium centers. In this compound, TFSA anions bridged two lithium ions through sulfonyl groups in a chelate and terminal coordination modes. The average Li−O distance in **5** was estimated to be 2.03 Å, which was shorter than that seen in compounds **3** and **4**, although the average Li−N distances of all three compounds were similar. Ordered arrays of lithium ions corresponding to ionic conduction paths were also confirmed in this compound. The closest intermolecular Li−Li distance was calculated to be 8.42 Å in compound **5**, which was longer than that seen in compound **2** (7.93 Å). These results indicate that structural control of ion conduction paths in MCs can be induced by controlling the molar ratio between the lithium salt and the small molecules as well as by altering the structures of the component molecules.

**Figure 9.**
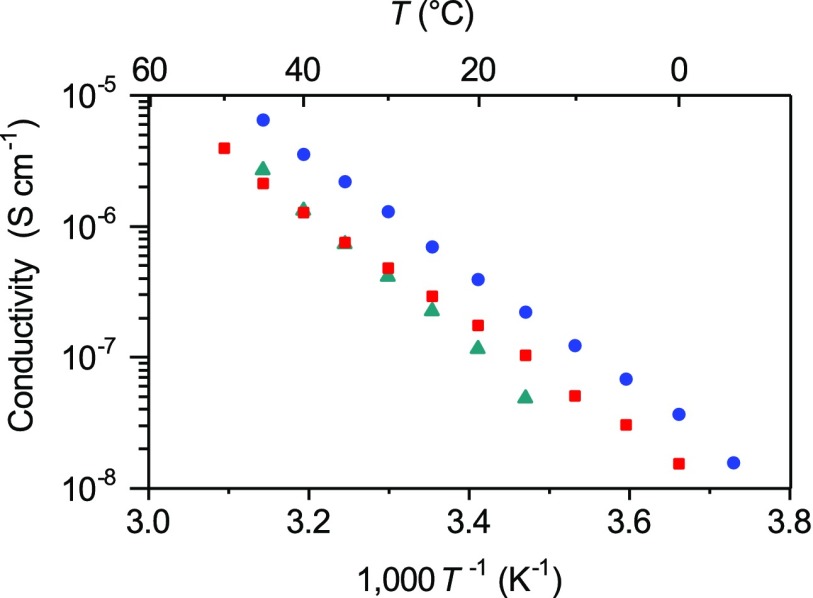
Ion conductivities of compounds **3–5** as a function of temperature (compound **3**: blue; compound **4**: green; compound **5**: red).

MCs **3–5** showed moderate ion conductivities, with values at 45°C being estimated to be 6.5 × 10^−6^, 2.7 × 10^−6^ and 2.0 × 10^−6^ S cm^−1^ for compounds **3**, **4** and **5**, respectively (Figure 9). Linear Arrhenius plots were observed for these MCs, as was seen for compound **2**. Moreover, the DSC curves of compounds **3–5** gave only one peak for melting. These observations indicate that ion conduction proceeded via the hopping mechanism through the continuous arrays of channel structures revealed by X-ray crystallography. The activation energies were calculated to be 0.87, 1.02 and 0.81 eV for compounds **3**, **4** and **5**, respectively. Importantly, the conductivities of compounds **3–5** exceeded that of compound **2**, where the channel structure showed a large chelate effect. The substitution of the tetradentate glyme units in compound **2** with the bidentate dimethoxybenzene frameworks enhanced the ion conductivities because of the decreased chelate effect. From the measurements of *t*_Li+_, high values of 0.9 were calculated for compounds **3** and **4**. Since the TFSA anions in compounds **3** and **4** behave as components of the ionic conduction paths, anion conduction was inhibited in these MCs as was seen in compound **2** resulting in selective lithium ion conductivity.

The structural comparison of compounds **3–5** suggests that shortening the Li–Li distances enhances lithium ion hopping through MCs with conduction paths, as does decreasing the strength of the Li−O interaction. Compound **3** had a considerably shorter Li–Li distance than compounds **4** and **5**, and **3** showed the highest ion conductivity. Since the lithium ion conductivity of compound **3** was improved when compared to compounds **4** and **5**, a short Li–Li distance can be seen to be an important factor for increasing lithium ion conduction.

## 4. MCs with diamine frameworks

**Figure 10.**
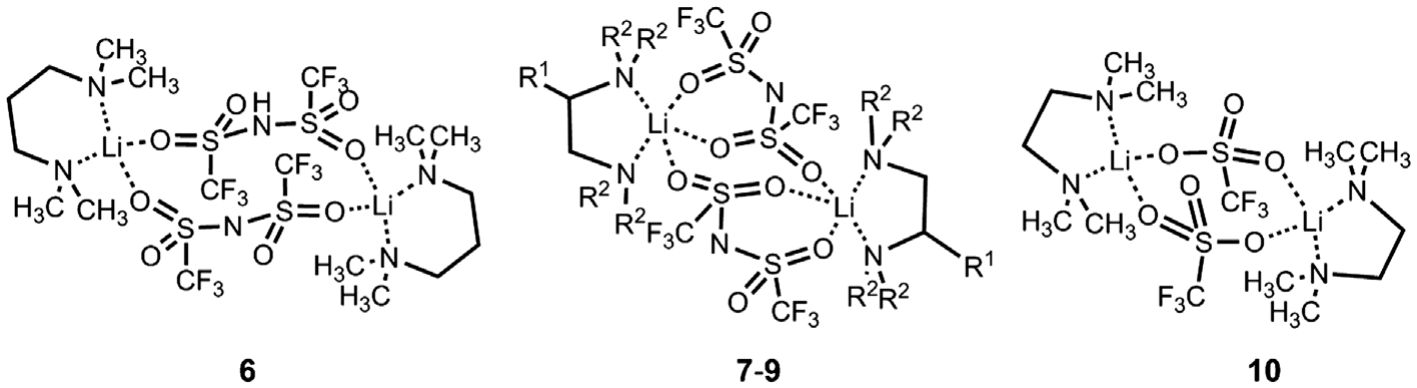
Molecular structures of compounds **6–10** (compound **7**: R^1^=CH_3_, R^2^=CH_3_; compound **8**: R^1^=H, R^2^=CH_2_CH_3_; compound **9**: R^1^=H, R^2^=CH_3_).

The obtained results showed that the structure of the conduction paths greatly affects the conductive properties of MCs. However, clear guidelines for the design of molecular crystalline electrolyte materials were not obtained because the fundamental relationship between the structure of the conduction paths and the lithium ion diffusivity in MCs was still unclear. Hence, we attempted to control the structure of the MCs systematically to clarify the influence of the structural features of the paths on the lithium ion conductivity. For this purpose, we focused on the use of diamines as components of the conduction paths because of their structural versatility. Diamines containing ethylenediamine units, R^1^_2_ NCHR^2^CH_2_ NR^1^_2_ (R^1^=CH_3_ or CH_2_CH_3_, R^2^=H or CH_3_), and propylenediamine frameworks, (H_3_C)_2_ NCH_2_CH_2_CH_2_ N(CH_3_)_2_ (TMPDA), were employed. Using these diamines, we synthesized the novel MCs [Li(TFSA)((H_3_C)_2_ NCH_2_CH_2_CH_2_ N(CH_3_)_2_)] (compound **6**), [Li(TFSA)((H_3_C)_2_NCH(CH_3_)CH_2_N(CH_3_)_2_)] (compound **7**) and [Li(TFSA)((H_3_CH_2_C)_2_NCH_2_CH_2_N(CH_2_CH_3_)_2_)] (compound **8**), and evaluated their conductivities [35] (Figure 10). We also measured the conductivities of [Li(TFSA)((H_3_C)_2_NCH_2_CH_2_N(CH_3_)_2_)] (compound **9**) [44] and [Li(CF_3_SO_3_)((H_3_C)_2_NCH_2_CH_2_N(CH_3_)_2_)] (compound **10**) [45], whose crystal structures have recently been reported.

**Figure 11.**
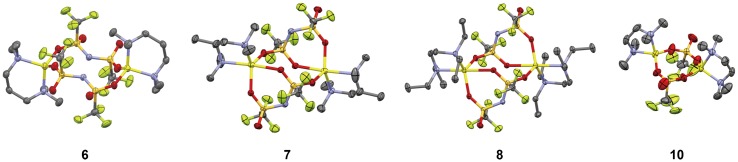
Crystal structures of compounds **6–8** and **10** determined from single-crystal X-ray diffraction study at -120 °C. (Li: yellow, C: gray, N: blue, O: red, F: green, S: dark yellow. Hydrogen atoms have been omitted for clarity.) (Adapted from [35] with permission from The Royal Society of Chemistry.).

Compound **6** was produced as a dilithium complex (Figure 11). The lithium centers of compound **6** were bridged by TFSA anions via the sulfonyl oxygen atoms to form 12-membered rings composed of lithium ions and TFSA anions. The lithium ions adopted a tetrahedral coordinate structure due to the chelate coordination of TMPDA and the bridging coordination of the two TFSA anions. The average Li–O and Li–N distances of compound **6** were determined to be 2.04 and 1.94 Å, respectively. The coordination environment of the lithium ions in compound **6** was similar to that seen in compound **10**, which has tetrahedral four-coordinate lithium centers bridged by OTf anions. The average length of the Li–O and Li–N bonds of compound **10** were also analogous with that of compound **6**, which were calculated to be 1.90 and 2.08 Å, respectively.

Compounds **7** and **8** were also yielded as dilithium complexes with lithium ions in trigonal bipyramidal structures (Figure 11). The structures of compounds **7** and **8** were almost identical to that of compound **9**, although the substituent groups in the diamine frameworks were different. In these compounds, three of the four sulfonyl groups of the TFSA anions formed interactions with the lithium centers. The average Li–O distances of compounds **7** and **8** were calculated to be 2.13 and 2.12 Å, while the average Li–N distances were estimated to be 2.12 and 2.14 Å, respectively. In compound **9**, these distances were calculated to be 2.09 Å for Li–O and 2.10 Å for Li–N.

**Figure 12.**
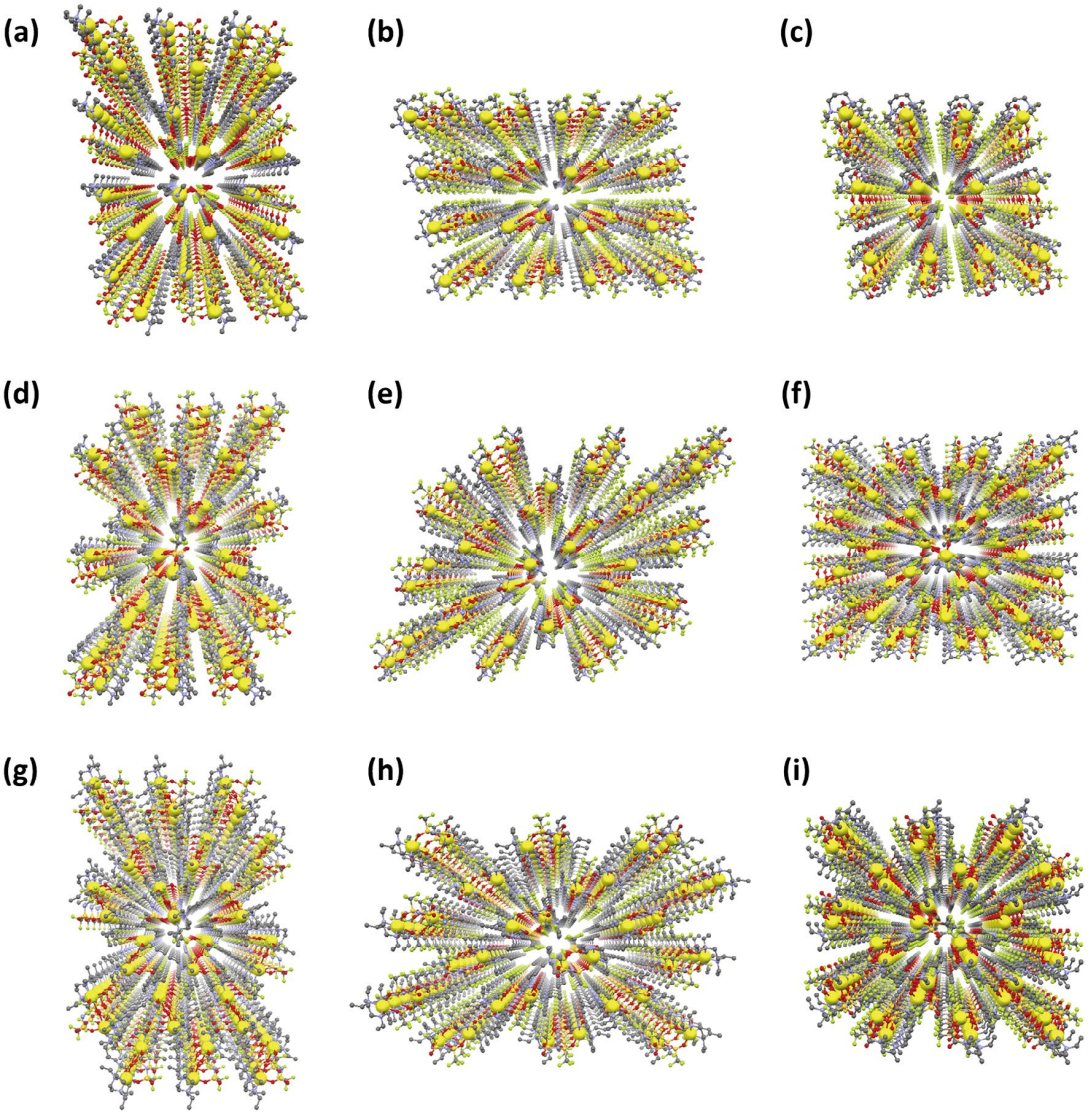
Ordered arrangement of lithium ions in compounds **6**–**8**. (a) compound **6**: along *a*-axis, (b) compound **6**: along *b*-axis, (c) compound **6**: along *c*-axis, (d) compound **7**: along *a*-axis, (e) compound **7**: along *b*-axis, (f) compound **7**: along *c*-axis, (g) compound **8**: along *a*-axis, (h) compound **8**: along *b*-axis, (i) compound **8**: along *c*-axis. (Li: yellow, C: gray, N: blue, O: red, F: green, S: dark yellow. Hydrogen atoms are omitted for clarity). (Adapted from [35] with permission from The Royal Society of Chemistry.).

The packing views of compounds **6**–**8** depict 3D ordered arrays of lithium ions in their crystal lattices similar to compounds **3–5**, showing the formation of ion conduction paths (Figure 12). The nearest intermolecular Li–Li distances for compounds **6**–**8** were estimated to be 6.65, 8.19 and 8.35 Å, respectively, which affected the lithium ion conductivity as mentioned above. In compound **9**, this distance was calculated to be 6.79 Å from the reported crystallographic data (CCDC 212577) deposited in the Cambridge Crystallographic Data Centre (CCDC) database. Since the CIF file of compound **10** was not found in the CCDC database, we performed an X-ray diffraction study of this compound to reveal a Li–Li distance of 6.89 Å.

The melting points of novel MCs **6–8** were estimated to be 71.5, 113.7 and 52.2 °C using DSC, respectively. Only one single peak attributed to melting was observed in the DSC curves of these MCs, which suggests that the 3D ordered arrangements of lithium ions revealed at −120 °C are maintained under ambient temperature.

**Figure 13.**
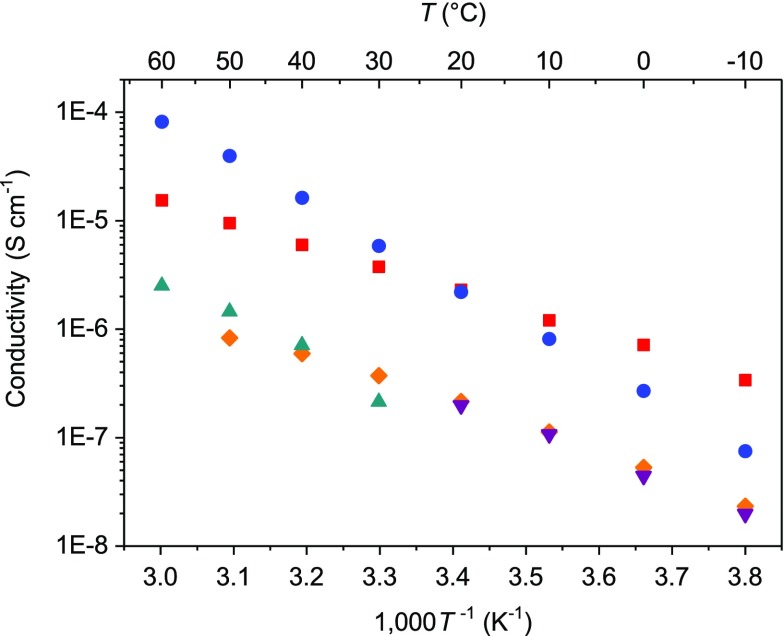
Ion conductivities of compounds **6–10** as a function of temperature. (Compound **6**: blue; compound **7**: orange; compound **8**: purple; compound **9**: red; compound **10**: green.) (Reproduced from [35] with permission from The Royal Society of Chemistry.).

Compounds **6**–**10** exhibited ion conductivities ranging from 10^−7^ to 10^−5^ S cm^−1^ at ambient temperature, and the linearity of their Arrhenius plots indicates that the ionic conduction occurred via an ion-hopping mechanism (Figure 13). The measurement of the *t*_Li+_ of compound **6** at 60 °C, where ion conductivity was sufficiently high for the measurement, gave a high *t*_Li+_ value of 0.95. The linearity of the Arrhenius plots and the high *t*_Li+_ numbers indicate that lithium ion hopping occurred selectively via the conduction paths in the obtained compounds.

The above-mentioned relationship between the crystal structures of compounds **6**–**10** and their conductive properties suggests that the value of the activation energy was affected by the molecular structure. The structural parameters of compounds **7**–**9** were almost identical, including their coordination geometries and numbers of lithium centers as well as the Li–O and Li–N bond lengths. These structural similarities seem to be reflected in the similar activation energies of compounds **7**–**9**, which were estimated to be 0.45, 0.49 and 0.41 eV, respectively. The relationship between the molecular structure and the activation energy can also be observed for compounds **6** and **10**. As remarked above, the lithium centers in compounds **6** and **10** had similar coordination environments comparable to average Li–O and Li–N distances, and the same coordination number. These structural similarities led compounds **6** and **10** to exhibit similar activation energies, calculated to be 0.76 and 0.71 eV, respectively. These results strongly support that the coordination environment of lithium ions, represented by the average distances between lithium ions and the coordination sites in the channel structure as well as the coordination number of the lithium centers, is a key factor in determining the activation energy for ion hopping through MCs.

The relationship between molecular arrangement and ion conductivity in MCs can also be seen by comparing compounds **7–9**. The Li–Li distances of compounds **7** and **8** were calculated to be 8.2–8.4 Å, whereas that of compound **9** was estimated to be 6.65 Å. Owing to steric repulsion derived from the increased bulkiness of (H_3_C)_2_ NCH(CH_3_)CH_2_ N(CH_3_)_2_ and (H_3_CH_2_C)_2_ NCH_2_CH_2_N(CH_2_CH_3_)_2_ compared to (H_3_C)_2_NCH_2_CH_2_N(CH_3_)_2_, compounds **7** and **8** had sparser molecular arrangements than compound **9**. On the other hand, compound **9** showed a conductivity that was approximately 10 times higher than those of compounds **7** and **8**. This result strongly suggests that a short intermolecular Li–Li distance is an important factor for enhancing the frequency of lithium ion hopping through the conduction paths.

However, compounds **6** and **10** showing similar structural parameters regarding the coordination environments of their lithium centers and their almost identical intermolecular Li–Li distances (6.65 Å for compound **6** and 6.89 Å for compound **10**), the ion conductivity of compound **6** was approximately 30 times higher than that of compound **10**. This difference in the ion conductivities is attributable to the number of vacant hopping sites. In these MCs, S=O groups without Li–O interactions are expected to act as vacant hopping sites. Compound **6** possessed four S=O groups in its structure capable of acting as vacant sites, whereas there were only two vacant S=O moieties in compound **10**. This result implies that the number of vacant hopping sites is also a key factor when attempting to enhance lithium ion diffusion.

These results provide fundamental guidelines for the design of molecular crystalline electrolytes with low activation energies and high ionic conductivities. The concept is simple: the activation energy can be reduced by weakening the interactions between the lithium ions and the channel structures, while ionic diffusion can be enhanced by shortening the intermolecular Li–Li distances and providing multiple vacant hopping sites.

MCs are easily obtained by a recrystallization process, which is the addition of organic compound to lithium salt at ambient temperature. It is a characteristic of molecular crystalline electrolytes that the synthetic conditions are mild compared to those of ceramic and glass electrolytes fabricated by calcination. Furthermore, the amounts of flammable organic substances in the molecular crystalline electrolytes are lower than those of polymer electrolytes and conventional liquid electrolytes. This indicates the high safety of the MC-based electrolytes. Moreover, the structural versatility of the supramolecular solids will be a powerful tool for a drastic improvement of ion conductivity. The structures of the conduction paths composed of supramolecular assemblies can be easily controlled by altering the component molecules. In fact, the ion conductivity of the MCs is comparable to that of PEO-based polymer electrolytes [46]. We also succeeded in the selective lithium ion conduction through MCs by using TFSA anions with coordination ability while the reported molecular crystalline electrolytes having noncoordinating anions such as BF_4_^−^ or AsF_6_^−^ show the relatively low *t*_Li+_ values due to the diffusion of these counter anions [24,25,28]. It is expected that development of molecular crystalline electrolytes by utilizing unique features of supramolecular solids creates new topics in the fields of the solid state ionics.

## 5. Conclusions

We synthesized novel MCs, consisting of LiTFSA and small molecules, which exhibited ion conduction paths. The MCs showed selective lithium ion conductivity in the solid state. The conduction paths consisted of ordered arrangements of channel structures, which were a result of the self-assembly of TFSA anions and small molecules with coordination sites. We also found that the modification of the component units of these MCs enabled precise, systematic structural control of the ionic conduction paths in the crystal structures. Based on these results, we clarified the relationship between the crystal structure and ion conductivity of the obtained MCs. The coordination environment of the lithium centers was a critical factor for determining the activation energy of ion diffusion. The magnitude of the ionic conductivity was influenced by the intermolecular Li–Li distances, which were affected by the molecular arrangement, and the number of vacant hopping sites, which was influenced by the molecular structure outside the lithium centers. These observations of the influence of supramolecular hierarchical structure on molecular ionics provide unconventional strategies that could open the door to the design of new solid electrolytes and thus the development of new molecular devices.

## Disclosure statement

No potential conflict of interest was reported by the author.

## Funding

The present review article was supported by JST-PRESTO ‘New Materials Science and Element Strategy’.
